# The effect of multi-component exercise on cognition function in patients with diabetes: A systematic review and meta-analysis

**DOI:** 10.1371/journal.pone.0304795

**Published:** 2024-06-20

**Authors:** Zhiyuan Sun, Hualei Liu, Min Yan, Haiqing Zeng, Yiping Hu, Xuewen Tian, Dewei Mao

**Affiliations:** 1 Qufu Normal University, Qufu, Shandong, China; 2 Shandong Sport University, Jinan, Shandong, China; Hamasaki Clinic, JAPAN

## Abstract

**Background:**

This meta-analysis investigated the influence of exercise on cognitive function in people living with diabetes.

**Methods:**

Stringent criteria for literature inclusion and exclusion were defined. Searches were conducted across four English databases to gather randomized controlled trials investigating exercise interventions for cognitive function in people living with diabetes. Outcome indicators from 1193 subjects across 12 articles were analyzed using RevMan 5.4 software.

**Results:**

Exercise intervention demonstrated the ability to mitigate cognitive decline in people living with diabetes, with a combined effect size (standardized mean difference) of 0.91, 95% CI: 0.28, 1.54, P < 0.00001. The intervention effect showed significant modulation by intervention content (I^2^ = 95%), intervention duration (I^2^ = 95%), intervention frequency (I^2^ = 95%), and intervention cycle (I^2^ = 96%). Among these factors, multi-component exercise, sessions >40 minutes, exercise frequency >4 times per week, and sustained exercise for >6 months were paramount, all with P < 0.05.

**Conclusion:**

Exercise intervention emerges as a viable strategy for delaying cognitive decline in people living with diabetes. Its efficacy is subject to modulation by various variables. Optimal intervention includes multi-component exercise, individual sessions lasting 40–60 minutes, exercising >4 times a week, and continuous exercise for over 6 months.

## Introduction

Diabetes is the predominant metabolic disorder globally, with projections estimating a staggering 1.3 billion individuals affected by 2050 [1]. Of significant concern is the potential disruption to glucose and lipid metabolism, leading to dysfunction in various bodily systems, including the brain, kidneys, liver, peripheral nervous system, and immune system [2]. Notably, people living with diabetes face a 1.5–2.0 times higher risk of experiencing cognitive decline, cognitive impairment, and dementia compared with people with non-diabetes [3]. The prevalence of cognitive dysfunction among people living with diabetes is an alarming 45% [4]. This rapid onset of cognitive impairment not only imposes a substantial economic burden on families and society but also puts considerable strain on national healthcare systems.

In light of the prolonged and challenging trajectory of diabetes-related cognitive impairment, the lack of an effective pharmaceutical intervention emphasizes the urgent need for alternative approaches. Concurrent medication usage poses heightened risks of side effects [5]. Within this context, exercise has become central to enhancing cognitive function in people with living diabetes. Moderate exercise can improve the cognitive function of the brain, but the results of related randomized controlled trials (RCTs) have been inconclusive. While several studies assert the cognitive benefits of exercise for people living with diabetes [6], conflicting results have cast doubt on the significance of exercise intervention [7], sparking controversy over its impact on cognitive function in this population. The research debate mainly focuses on two aspects: the first is to improve the process and validity with the applicable population, and the second is the exercise program.

Multi-component exercise refers to the intervention of two or more types of exercise [8], such as the combination of aerobic exercise, strength or resistance training, balance or coordination training, flexibility training, and other exercise methods. In evidence-based medicine research, the standardized mean change score (SMCS) of cognitive assessment before and after exercise intervention was used as the effect value, and multi-component exercise (SMCS = 0.59) was found to improve the cognitive ability of healthy elderly individuals more than aerobic exercise (SMCS = 0.41) [9]. Moreover, the intricacies of exercise intervention necessitate careful consideration of adjustment variables, such as exercise mode, cycle, and frequency. Notably, the quantity and duration of exercise exert influence on cerebral blood flow and oxygen content, potentially elucidating the biological mechanisms underpinning the cognitive effects of exercise through cortical activity [10]. Despite this, a definitive understanding of the dose–effect relationship between regulatory variables and cognitive function in diabetes remains elusive. Therefore, to facilitate more precise insights into the impact of exercise on cognitive health, this study investigated the potential benefits of exercise intervention in enhancing cognitive function among people living with diabetes. Addressing previous research gaps, the investigation aimed to furnish robust evidence, elucidating sources of heterogeneity among studies, and advancing the comprehension of the nuanced interplay between exercise and cognitive well-being in people living with diabetes.

## Research methods

This study meticulously followed the procedural frameworks outlined in the Cochrane Handbook for Systematic Reviews and Meta-Analysis, along with strict adherence to the PRISMA (Preferred Reporting Items for Systematic Reviews and Meta-Analysis) statement [11]. The review protocol was registered in the international prospective register of systematic reviews (PROSPERO), with the identification title, “Effects of exercise therapy on cognitive function in person with diabetes: A systematic review of a randomized controlled trial”(CRD42024502236).

### Literature retrieval strategy

Two researchers meticulously crafted a comprehensive literature search strategy, using PubMed, EBSCOhost, Web of Science, and Elsevier databases. The search encompassed the entire period of these databases from their inception up to December 2023, and final retrieval was conducted on December 23, 2023. The search keywords, detailed in Table 1, were strategically organized. Internally, each set of search keywords was linked with “OR,” and across sets, “AND” was employed as the connecting element, using Boolean operations for integrated retrieval. To further enhance inclusivity, manual retrieval was undertaken to trace and scrutinize the references of the included literature. This rigorous approach aimed to ensure the thoroughness and comprehensiveness of the assembled literature corpus.

**Table 1:** The search terms of the literature included in this study.

Group	Subject word
1	exercise, resistance strength, baduan jin, aerobic exercise, tai chi, qi gong, liu zi jue, wu qin xi, sports, physical activity, training, balance Pilates fitness, walking, aquatic, swimming, climbing, dance cycling, yoga, multi-component exercise
2	Cognition, mild cognitive impairment, mild neurocognitive disorder, memory disorder, memory impairment, memory decline early-stage dementia, cognitive decline, cognition dysfunction, mental deterioration
3	type 2 diabetes, diabetes, type 1 diabetes, diabetes mellitus, diabetic, experimental diabetic, hyperglycemia and glucosuria, diabetes glycosuria, glycuresis

### Inclusion and exclusion criteria

Adhering to the Cochrane meta-analysis PI-COS principle, this study systematically considered four pivotal factors: participants, intervention measures, research results, and research design.

Inclusion criteria were meticulously outlined as follows:1) Participants: Individuals diagnosed with diabetes, with the condition defined in accordance with standardized guidelines, such as those set forth by the American Diabetes Association, the World Health Organization, or the International Diabetes Federation. 2) Intervention measures: Exercise intervention was administered, using a control group. In the experimental group, exercise intervention constituted the exclusive interventional measure, with flexibility in the choice of exercise method. 3) Outcome indicators: Cognitive function served as the primary outcome, which was assessed through established measurement tools, including the Montreal Cognitive Scale (MoCA) and Mini-Mental State Examination (MMSE). 4) Study design: The study encompassed clinical RCTs published in English. Notably, there was no significant disparity between the experimental and control groups.

Exclusion criteria were defined as follows: 1) Studies diverging from the meta-analysis theme, specifically those involving participants without diabetes or lacking exercise intervention. 2) Instances where outcome indicator data were incomplete, impeding the calculation of the standardized mean difference (SMD) score post intervention. 3) Redundant or overlapping articles were systematically excluded. 4) Reviews, editorials, animal experiments, or dissertations were categorically excluded to ensure the specificity and relevance of the selected studies.

### Literature screening and data extraction

The gathered literature underwent initial processing using the literature management software Endnote X9 to eliminate duplicates. Subsequently, a rigorous screening process was conducted by two researchers in an independent double-blind manner, adhering to predefined inclusion and exclusion criteria. Discrepancies in the inclusion decision were resolved through mutual comparison, and if unresolved, third-party adjudication was sought. For the literature meeting the criteria, a structured extraction table was created. Information extraction was carried out independently by the two researchers, focusing on the following three aspects: 1) Basic information: This encompassed details such as the first author, publication year, and country of origin. 2) Intervention characteristics: Comprehensive details were extracted, including sample size, characteristics of the study population (age, gender), specifics of the intervention (content, time, frequency, cycle), and a concise program description. 3) Outcome indicators: The cognitive function of people living with diabetes was evaluated using commonly employed neuropsychological tests. The primary outcome of this study was the observed change in cognition throughout the entire intervention period, with the secondary outcome being the cognitive scores post intervention. Any discrepancies or uncertainties in the extraction process were systematically addressed through discussion and consensus.

### Evaluation of bias in the included literature

The assessment of bias in the included literature used the Cochrane Collaboration’s RCT bias evaluation tool [12], covering the following criteria: 1) generation of random sequences, 2) allocation hiding, 3) blindness of implementers and participants, 4) blind method of result evaluation, 5) integrity of result data, 6) selective reporting, and 7) other sources of bias—bias risk evaluation was conducted independently by two researchers, and any discrepancies were resolved through negotiation or discussion with a third researcher. Each criterion was categorized as low bias risk, bias uncertainty, or high bias risk. Based on the assessment results, the quality of the included literature was stratified into three grades: 1) Grade A: met the low risk criteria for four or more items, 2) Grade B: met the low risk criteria for 2 or 3 items, and 3) Grade C: met the low risk criteria for one or no items.

### Statistical methods

RevMan 5.4 was used for the comprehensive analysis of outcome indicators derived from the included literature. SMD and 95% confidence interval (95% CI) were the effect scales for the combined effect sizes. Initially, the homogeneity test, using a significance level (α) of 0.1, was conducted to assess the heterogeneity of the literature, employing the Chi-square test. A P-value less than α indicated the presence of heterogeneity among the studies, while homogeneity was inferred otherwise. Simultaneously, I^2^ was used for a quantitative evaluation of inter-study heterogeneity, following Cochrane Handbook criteria. An I^2^ value of 0 denoted no heterogeneity, and when I^2^ was <50%, the fixed-effect model was selected for analysis. In cases where I^2^ >50%, the random-effect model was applied. High heterogeneity triggered subgroup analysis to identify its source. Sensitivity analysis was conducted to examine the stability of the results. Subsequently, Egger’s test was used to assess publication bias for each index. A funnel plot facilitated the analysis of publication bias. Meta-analysis was conducted on cognitive changes throughout the entire intervention period and cognitive scores post intervention in both the experimental and control groups. Consistency between the included literature and the test units was observed. Mean difference served as the effect size, with the SMD calculated. A 95% confidence interval was determined, and significance was established at P < 0.05.

## Results

### Literature search results

**Fig 1:**
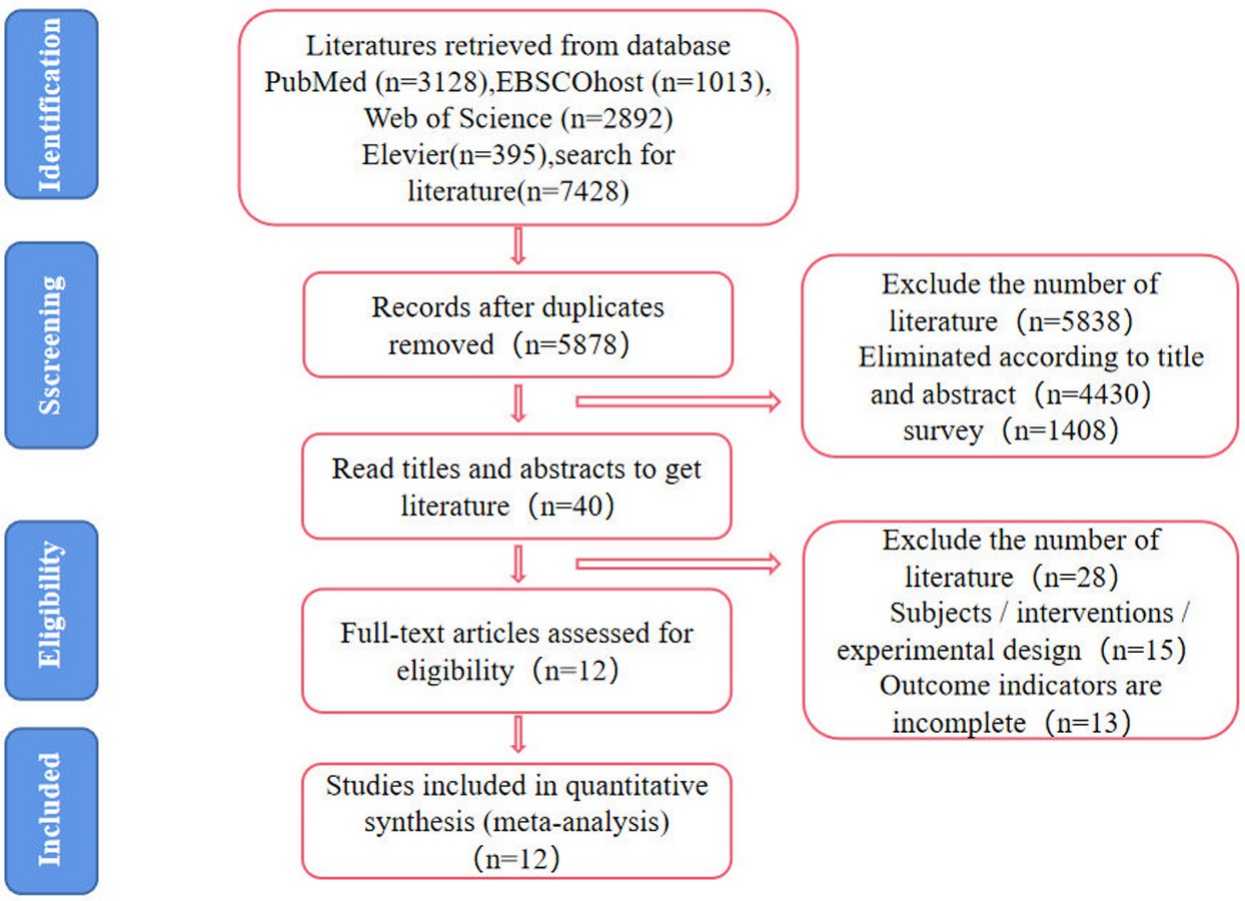
Flow diagram of study selection procedure.

A comprehensive literature database search yielded 7428 articles, subsequently screened for duplicates using EndNote X9. After removing duplicates, 5878 articles remained. The titles and abstracts of these articles were meticulously reviewed to eliminate irrelevant entries, leading to the exclusion of 5838 articles. The remaining 40 articles underwent a thorough full-text examination, resulting in the exclusion of 28 articles. Ultimately, 12 articles, all in English (Fig 1), met the strict inclusion criteria for the meta-analysis.

### Basic characteristics of the included literature

This study includes a total of 12 articles, involving a comprehensive sample size of 1193 individuals, with males constituting approximately 48% of the cohort. All participants had a diagnosis of diabetes. The literature under consideration provided details on the sample sizes for both the experimental (593) and control (600) groups. Cognitive function was assessed using MMSE and MoCA. In the experimental group, interventions varied: 4 studies employed aerobic exercise, 2 studies used resistance exercise, 4 studies implemented multi-mode exercise (a combination of 2 or more exercise modes), and 2 studies incorporated physical and mental exercise interventions. Regarding exercise parameters, 8 studies advocated an exercise frequency of ≤3 days per week, while 4 studies endorsed a frequency >3 days per week. Additionally, 4 studies recommended an exercise cycle of ≤12 weeks, 5 studies suggested a cycle >12 but not >24 weeks, and 2 studies extended the cycle beyond 24 weeks. Moreover, 3 studies proposed an exercise time of ≤40 minutes per session, while 9 studies advocated a duration exceeding 40 minutes per session. In contrast, the control group predominantly underwent routine nursing and health education interventions. For a comprehensive overview of the literature’s characteristics, refer to Table 2.

**Table 2:** Baseline characteristics of the 12 included studies.

Author (year/ country)	Subjects		Intervention measures						Cognitive outcome
	male/all	Age (years)	Exercise categories	Intervention exercise	Frequency (times/week)	Time (min/week)	Duration (week)	Therapy for control group	
**Yannan Chen et al. (2023/China) [54]**	100/218	67.5 ± 4.99	Mind-body exercise	24-form tai chi chuan training	3	180	24	Workshops	MoCA
**Tawatchai Ploydanget al. (2023/America) [50]**	12/33	68.9 ± 3.7	Aerobic exercise	Nordic walking in a swimming pool	3	120	12	Maintained daily activities	MoCA, MMSE
**Chidananda Kaligal et al. (2023/India) [55]**	37/50	52.5 ± 8.7	Mind-body exercise	Yoga	3	-	12	Maintained daily activities	MoCA
**Joyla A. Furlano et al. (2023/Canada) [56]**	12/24	68.2 ± 6	Resistance training	Resistance training	3	180	24	Balance, stretching, and range-of-motion exercises	Stroop Test et al
**Nafiseh Ghodrati et al. (2022/Canada) [49]**	NA/21	58.8 ± 1.5	Multi-component exercise	warm-up, aerobic exercise, resistance workouts, balance exercise, cooldown	3	195	12	Maintained daily activities	MoCA
**Martinet-Village et al. (2021/America) [57]**	50/1033	87 ± 4	Multi-component exercise	Included individualized supervised progressive resistance, balance, and walking exercises.	5–7	≥200	-	Usual-care	MMSE
**Edgardo Molina-Sotomayor et al. (2021/Spain) [58]**	NA/76	64–78	Aerobic exercise	Walking	3	180	16	Maintained daily activities	MMSE
**Yutaro Yamamoto et al. (2021/Japan) [51]**	28/53	73.2 ± 2.6	Resistance training	Bodyweight resistance training with elastic bands exercises	7	105	48	Maintained daily activities	MMSE
**Edgardo Molina-Sotomayor et al. (2020/America) [59]**	NA/107	72.3 ± 3.7	Aerobic exercise	Walking	3	180	24	Maintained daily activities	MMSE
**Yi Zhu (2018/China) [52]**	24/60	70.3 ± 6.7	Aerobic exercise	Aerobic dance	3	105	12	Maintained daily activities	MoCA
**Mark A. Espeland et al. (2017/America) [60]**	281/415	70–89	Multi-component exercise	Walking, strength, flexibility, and balance training	3–4	≥150	96	Workshops and upper extremity stretching and flexibility exercises	3MSE
**M. L. Callisaya (2017/Australia) [53]**	26/50	65.3 ± 5	Multi-component exercise	An aerobic component of approximately 30 min and 30 min of moderate-to high-intensity PRT	2	120	24	A stretching/gentle movement	Victoria Stroop test et al

### Risk bias assessment of included literature

**Fig 2:**
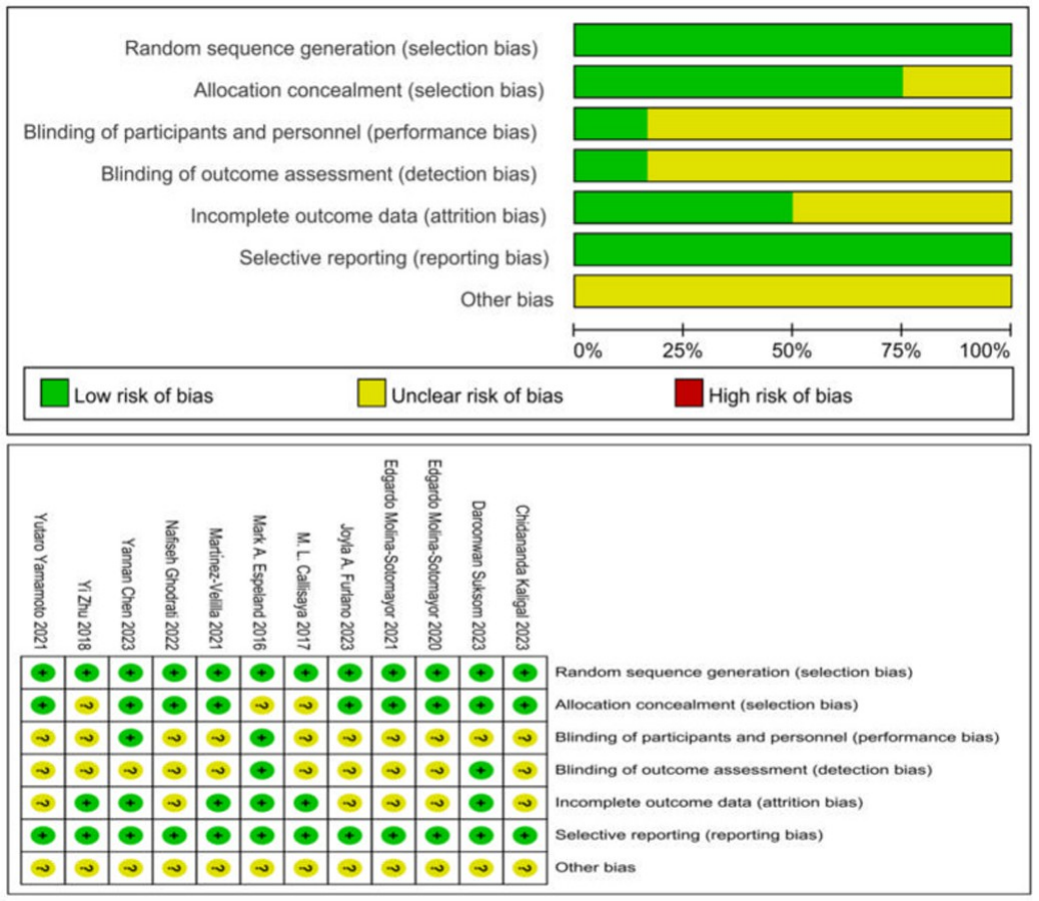
Analysis of the risk of bias in accordance with the Cochrane collaboration guidelines.

The authors used the Cochrane risk of bias assessment tool to evaluate the quality of the mentioned literature. The third author resolved any discrepancies, culminating in the final assessment of literature quality. According to the quality evaluation criteria, as illustrated in Fig 2, four articles met the criteria for low risk across four or more items, while eight articles showed low risk across two to three items. The resulting evaluation categorized four articles as Grade A and eight articles as Grade B.

### Meta-analysis results

#### Effect of exercise intervention on cognitive function in people living with diabetes

**Fig 3:**
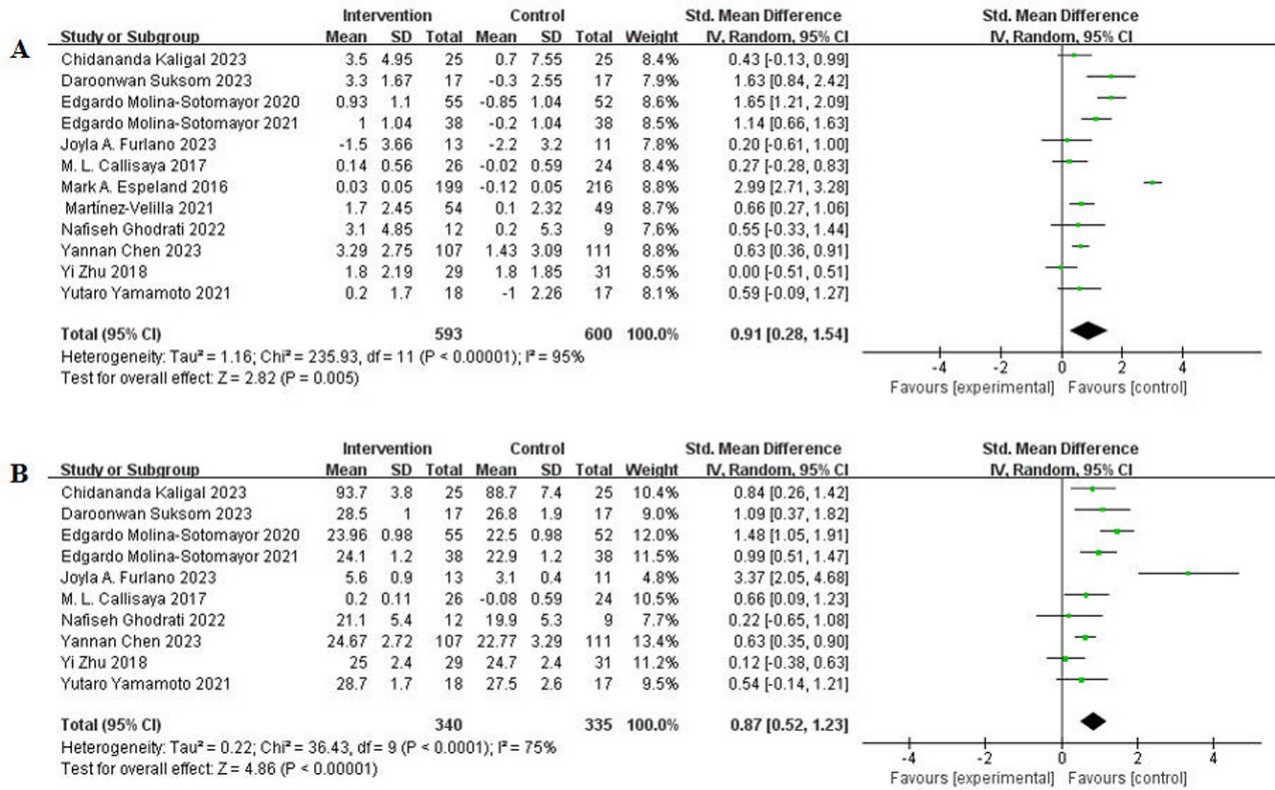
Forest plots for effect of exercise therapy on cognition of patients with diabetes: (A) The change of cognition throughout intervention duration. (B) Post-intervention cognitive scores.

The selected outcome indicators for assessing the overall impact of exercise intervention on the cognitive function of people living with diabetes included cognitive changes during the intervention period (12 articles) and post-intervention cognitive evaluation results (10 articles) in each study (see Table 3 for the included studies). In the 12 studies, the primary outcome indicator was the enhancement of cognitive function, and these studies reported on the association between exercise intervention and cognition in people living with diabetes. The heterogeneity test showed significant heterogeneity among the studies (I^2^ = 95%, >50%, P < 0.00001). Using the random-effect model for result combination, the synthesized effect size (SMD) was 0.91, with a 95% CI of 0.28–1.54, P < 0.00001 (see Table 4). The forest plot displayed the 95% CI horizontal line for the effect of exercise on the cognitive function of people living with diabetes, positioned to the right of the null line (see Fig 3A). These findings indicate the effectiveness of exercise intervention in enhancing the cognitive function of people living with diabetes. In the subset of 10 studies using cognitive scores post intervention as the outcome index, the heterogeneity test showed significant heterogeneity (I^2^ = 75%, >50%, P < 0.0001). The random-effect model was used for result synthesis, yielding a combined SMD of 0.87, with a 95% CI of 0.52 to 1.23, P < 0.00001. The forest plot depicted the 95% CI horizontal line for the effect of exercise on cognitive function in people living with diabetes, situated to the right of the null line (see Fig 3B). These results affirm the efficacy of exercise intervention in improving the cognitive function of people living with diabetes.

**Table 3:** Relative outcomes of the 12 included studies.

Study	Sample size (N) Intervention/Control	Change of cognition throughout intervention duration			Post-intervention cognitive scores (mean[SD])		
		Intervention (mean [SD])	Control (mean[SD])	SMD (95%CI)	Intervention (mean [SD])	Control (mean [SD])	SMD (95%CI)
**Martínez-Velilla et al. (2021)**	54/49	1.7 (2.45)	0.1 (2.32)	0.66 (0.27, 1.06)			
**Mark A. Espeland et al. (2016)**	199/216	0.03 (0.05)	−0.12 (0.05)	2.99 (2.71, 3.28)			
**Yannan Chen et al. (2023)**	107/111	3.29 (2.75)	1.43 (3.09)	0.63 (0.36, 0.91)	24.67 (2.72)	22.77 (3.29)	0.63 (0.35, 0.90)
**Daroonwan Suksom et al. (2023)**	17/17	3.3 (1.67)	−0.3 (2.55)	1.63 (0.84, 2.42)	28.5 (1)	26.8 (1.9)	1.09 (0.37, 1.82)
**Edgardo Molina-Sotomayor et al. (2020)**	55/52	0.93 (1.1)	−0.85 (1.04)	1.65 (1.21, 2.09)	23.96 (0.98)	22.5 (0.98)	1.48 (1.05, 1.91)
**Edgardo Molina-Sotomayor et al. (2021)**	38/38	1 (1.04)	−0.2 (1.04)	1.14 (0.66, 1.63)	24.1 (1.2)	22.9 (1.2)	0.99 (0.51, 1.47)
**Chidananda Kaligal et al. (2023)**	25/25	3.5 (4.95)	0.7 (7.55)	0.43 (−0.13, 0.99)	93.7 (3.8)	88.7 (7.4)	0.84 (0.26, 1.42)
**Nafiseh Ghodrati et al. (2022)**	12/9	3.1 (4.85)	0.2 (5.3)	0.55 (−0.33, 1.44)	21.1 (5.4)	19.9 (5.3)	0.22 (-.65, 1.08)
**Joyla A. Furlano et al. (2023)**	13/11	−1.5 (3.66)	−2.2 (3.2)	0.20 (−0.61, 1.00)	5.6 (0.9)	3.1 (0.4)	3.37 (2.05, 4.68)
**Yutaro Yamamoto et al. (2021)**	18/17	0.2 (1.7)	−1 (2.26)	0.59 (−0.09, 1.27)	28.7 (1.7)	27.5 (2.6)	0.54 (−.14, 1.21)
**Yi Zhu 2018**	29/31	1.8 (2.19)	1.8 (1.85)	0.00 (−0.09, 1.27)	25.0 (2.4)	24.7 (2.4)	0.12 (−.38, 0.63)
**M. L. Callisaya2017**	26/24	0.14 (0.56)	−0.02 (0.59)	0.27 (−0.28, 0.83)	0.20 (0.56)	−0.08 (0.59)	0.66 (0.09, 1.23)

**Table 4:** The overall effect test of exercise intervention on cognitive function in patients living with diabetes.

	Documents quantity	Sample size	Homogeneity-testing			Two-tail test		SMD	95%CI
			X^2^	P	I^2^	Z	P		
**Random data effect model**	12	1193	235.93	< 0.00001	95	2.82	= 0.005	0.91	(0.28, 1.54)

### Sensitivity analysis and publication bias

**Fig 4:**
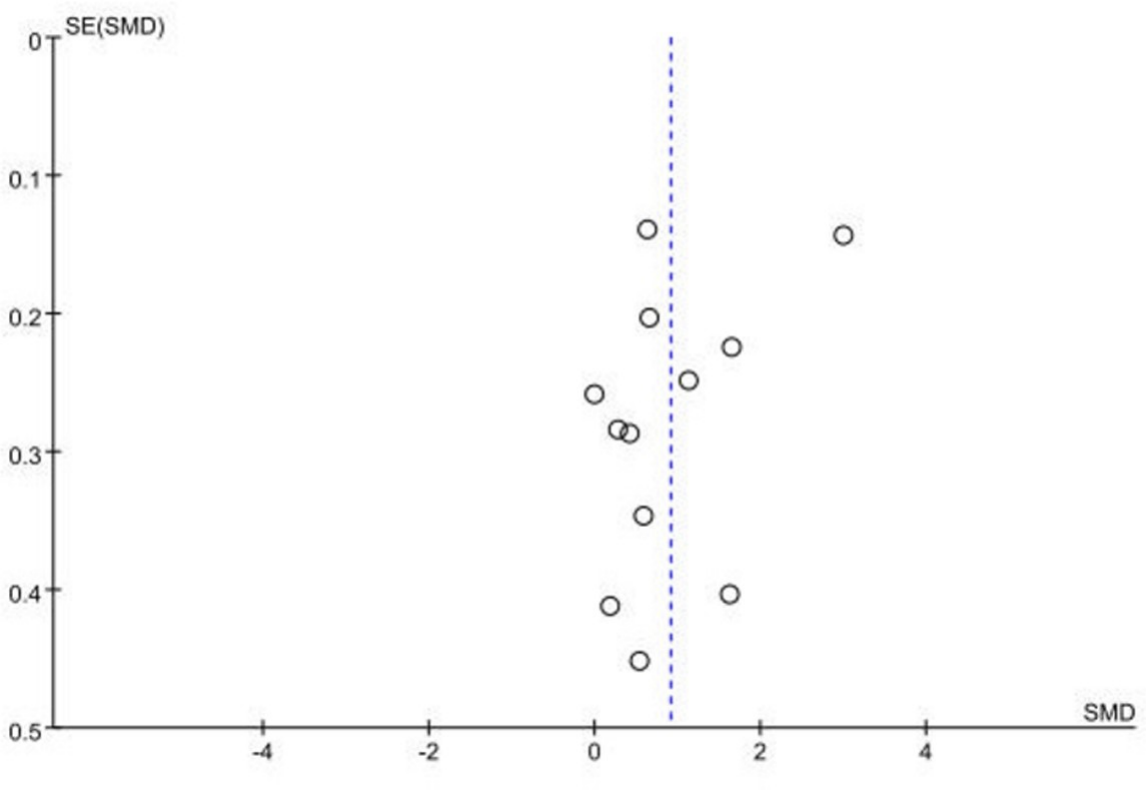
Funnel plot of publication bias.

In the sensitivity analysis, the combined outcomes of the meta-analysis using the random-effects model remained consistent with those derived from the fixed-effects model or the primary findings of the study with the lowest literature quality score. This consistency highlights the stability of the combined effect (SMD) of exercise intervention on cognitive function. To scrutinize potential publication bias, the customary practice involves generating and analyzing funnel plots for meta-analyses comprising more than 10 studies. Fig 4. shows a commendably symmetrical distribution across all studies. Subsequently, the Egger test (Table 5), with a P-value of 0.18 (> 0.05), suggests the absence of significant publication bias.

**Table 5:** Egger test results.

Std_Eff	Cofe	Std.Eff	t	p>|t|	(95%Conf.Interval)
**Slope**	2.310055	0.8102714	2.85	0.017	(0.5046578,4.115452)
**Bias**	-5.083136	3.514321	-1.45	0.179	(-12.91353,2.74726)

### Subgroup analysis

#### Effects of different exercise modes on cognitive function in people living with diabetes

**Fig 5:**
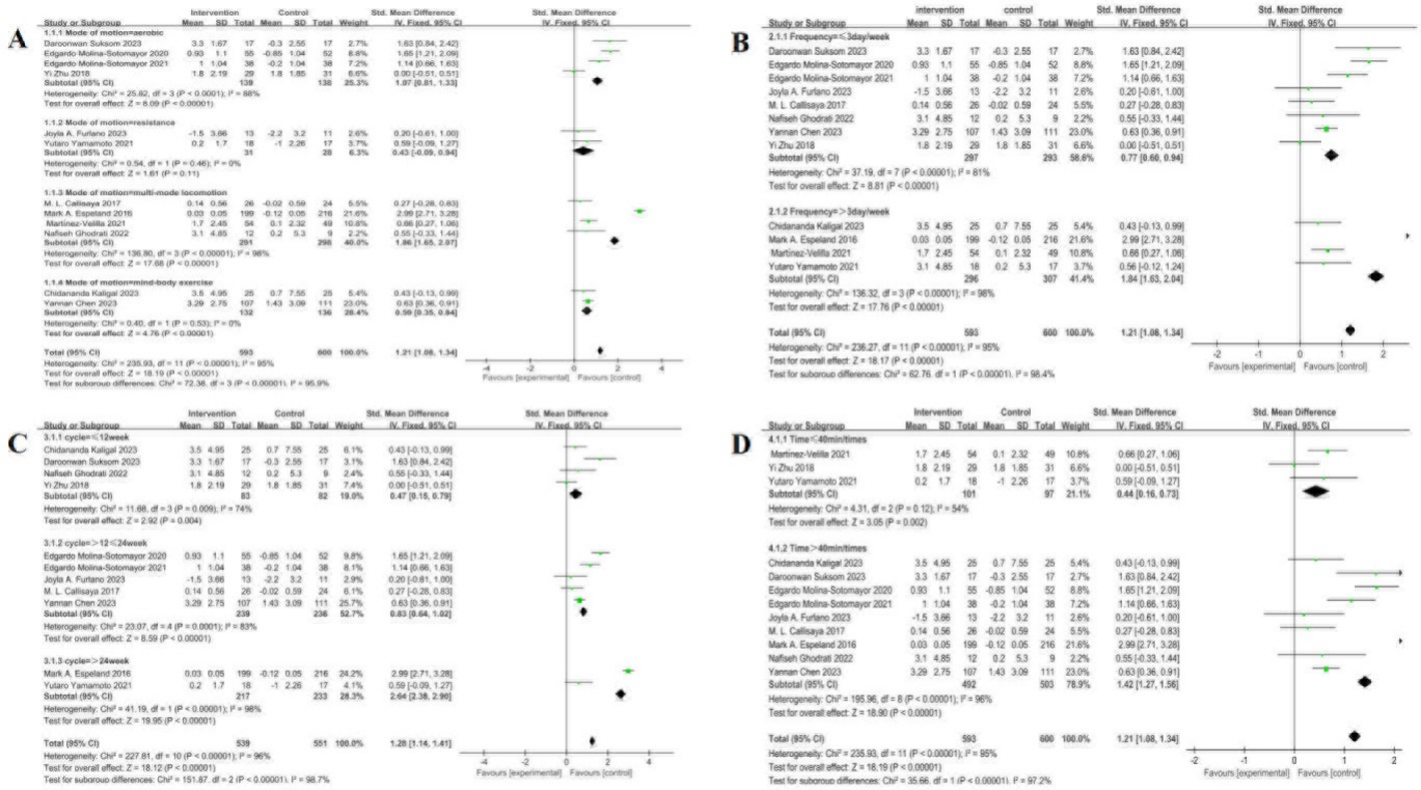
The forest map of the effect of exercise-related regulation on the cognition of diabetic patients: (A) Exercise form (B) Exercise frequency (C) Exercise time (D) Exercise duration.

A total of 12 studies, involving 1193 subjects (Table 6), were included in this cohort. Subgroup analysis was conducted meticulously across four distinct forms of exercise: aerobic exercise, resistance exercise, physical and mental exercise, and multi-component exercise (see Fig 5A). Notably, the four categories showed a tendency toward high heterogeneity in effect size differences (I^2^ = 95%), emphasizing the significant regulatory impact of intervention content on overall efficacy. Specifically, the heterogeneity test for the aerobic exercise subgroup showed X^2^ = 25.82, I^2^ = 88%, P < 0.0001, with a combined effect size (SMD) of 1.07, 95% CI: 0.81, 1.33, P < 0.00001—indicating a statistically significant difference. In contrast, the heterogeneity test for the resistance exercise subgroup revealed X^2^ = 0.54, I^2^ = 0%, P = 0.46, with a non-significant combined effect size (SMD) of 0.43, 95% CI: −0.09, 0.94, P = 0.11. The multi-component exercise subgroup showed substantial heterogeneity (X^2^ = 136.80, I^2^ = 98%, P < 0.00001) and a significant combined effect size (SMD) of 1.86, 95% CI: 1.65, 2.07, P < 0.00001. Similarly, the physical and mental exercise subgroup showed minimal heterogeneity (X^2^ = 0.40, I^2^ = 0%, P = 0.53) and a statistically significant combined effect size (SMD) of 0.59, 95% CI: 0.35, 0.84, P < 0.0001. Results indicate that the impact of multi-component exercise was the most pronounced.

**Table 6:** The moderating effect test of exercise intervention on cognitive function in people living with diabetes.

Regulated variable	Homogeneity-testing			Category	SMD	95%CI	Two-tail test		Documents quantity	Sample size
	X^2^	P	I^2^/%				Z	P		
**Means of intervention**	235.93	< 0.00001	95	Aerobic exercise	1.07	(0.81, 1.33)	8.09	< 0.00001	4	277
				Resistance exercise	0.43	(−0.09, 0.94)	1.61	0.11	2	59
				Multi-component exercise	1.86	(1.65, 2.07)	17.68	< 0.00001	4	589
				Mind-body exercise	0.59	(0.35, 0.84)	4.76	< 0.00001	2	268
**Frequency of intervention**	236.27	< 0.00001	95	≤3 times/week	0.77	(0.60, 0.94)	8.81	< 0.00001	8	590
				>3 times/week	1.89	(1.68, 2.09)	17.89	< 0.00001	4	603
**Intervention cycle**	227.81	< 0.00001	96	≤12 weeks	0.47	(0.15, 0.79)	2.29	0.004	4	165
				>12 & ≤24 weeks	0.83	(0.64, 1.02)	8.59	< 0.00001	5	475
				>24	2.64	(2.38, 2.90)	19.95	< 0.00001	2	450
**Intervention time**	235.93	< 0.00001	95	≤40 min/times	0.44	(0.16, 0.73)	3.05	0.002	3	198
				>40 min/times	1.42	(1.27, 1.56)	18.90	< 0.00001	9	995

#### Effect of exercise frequency on cognitive function in people living with diabetes

A comprehensive analysis involved a cohort of 12 studies with 1193 participants (Table 6). Stratifying exercise frequency into two categories: ≤3 days per week (inclusive of 3 days) and >3 days per week revealed significant heterogeneity in effect size, denoted by I^2^ = 95%. This observation highlights the substantial regulatory impact of exercise frequency on intervention outcomes (see Fig 5B). The heterogeneity examination for weekly exercise ≤3 days showed X^2^ = 37.19, I2 = 81%, P < 0.00001, with a combined effect size (SMD) of 0.77, 95% CI: 0.60, 0.94, P < 0.00001—indicating a statistically significant difference. Meanwhile, the heterogeneity test for weekly exercise >3 days showed X^2^ = 130.54, I^2^ = 98%, P < 0.00001, with a combined effect size (SMD) of 1.84, 95% CI: 1.68, 2.09, P < 0.00001, reinforcing a statistically significant distinction.

#### Effect of exercise cycle on cognitive function in people living with diabetes

In this cohort, 11 studies with a total of 1090 subjects were included (Table 6). The exercise duration was categorized into three groups: ≤12 weeks, >12 weeks and ≤24 weeks, and >24 weeks. A high degree of heterogeneity in the effect size difference among the three groups was observed (I^2^ = 96%), highlighting the substantial regulatory impact of the exercise cycle on intervention outcomes (see Fig 5C). Specifically, the heterogeneity test for people living with diabetes engaging in exercise lasting ≤12 weeks showed X^2^ = 11.68, I^2^ = 74%, P = 0.009. Similarly, for patients living with diabetes involved in exercise lasting >12 weeks and ≤24 weeks, results of the heterogeneity test were X^2^ = 23.07, I^2^ = 83%, P = 0.0001. Notably, in people living with diabetes with an exercise duration >24 weeks, the heterogeneity test showed X^2^ = 41.19, I^2^ = 98%, P < 0.00001. The combined effect SMD was 2.64 (95% CI: 2.38, 2.90), indicating a highly significant difference (P < 0.00001). This highlights that the impact of an exercise cycle >24 weeks was the most pronounced.

#### Effect of exercise time on cognitive function in people living with diabetes

A cohort of 12 studies, involving a total of 1193 subjects, was examined (Table 6). The temporal dimension of exercise sessions was categorized into two groups: individual sessions lasting ≤40 minutes and individual sessions >40 minutes. Notably, a significant degree of heterogeneity, reaching I^2^ = 95%, was observed between the two groups, emphasizing the crucial role of exercise duration in modulating the intervention effect (see Fig 5D). Upon conducting a heterogeneity test for individual sessions lasting ≤40 minutes, the results showed X^2^ = 4.31, I^2^ = 54%, P = 0.12. In contrast, results of the heterogeneity test for individual sessions >40 minutes were X^2^ = 195.96, I^2^ = 96%, P < 0.00001. These findings emphasize the substantial impact of exercise duration on the observed differences in intervention effects.

## Discussion

### Overall effect analysis of exercise on cognitive function in people living with diabetes

The rising prevalence of diabetes, especially the increase in type 2 diabetes (T2DM) [13], is progressively linked to changes in lifestyle factors such as dietary habits, overweight conditions, and sedentary behavior [14]. Simultaneously, a parallel trend in the growth of the population is observed in dementia cases [15]. Obviously, the combined rate of diabetes and dementia is higher than expected. Epidemiological studies have shown that people living with diabetes have an increased risk of mild cognitive impairment and dementia [16]. Scientific studies suggest that regular exercise contributes to improvements in cerebral blood volume [17], vascular reactivity [18], synaptic/neural plasticity, neurogenesis, and the regulation of nutritional factors [19]. Furthermore, exercise has a positive influence on brain structure and function, influencing behavioral development and mitigating disease risk.

This study, adopting an evidence-based clinical approach, examined the impact of exercise on cognitive function in people living with diabetes. The findings unequivocally indicate that exercise significantly slows down the decline in cognitive function among people living with diabetes [20]. Compared with recently published studies [20–22], this study was novel in that the included studies were all RCTs that analyzed only the evidence of the impact of physical exercise on cognitive performance in older patients living with T2DM. In addition, a larger sample of literature was included.

The complexities of cognitive impairment in people living with diabetes are multifaceted, involving structural and functional alterations in the brain. Shared pathological foundations with neurodegenerative diseases further contribute to this complexity. As a non-pharmacological intervention for preventing and addressing cognitive decline, the biological mechanisms supporting the enhancement of cognitive function through exercise have been substantiated by an extensive body of clinical and animal experiments. Advancing age in people living with diabetes is accompanied by a gradual decline in cardiovascular function, leading to reduced resting cerebral blood flow [23]. Exercise, as a modality, plays a crucial role in promoting cerebral blood circulation and orchestrating redistribution, concurrently amplifying antioxidant effects through increased enzyme activity and proinflammatory cytokines [24]. Imaging studies have also demonstrated that exercise has the ability to increase the overall volume of blood flowing through the brain per unit time [25]. However, researchers have suggested that improvement in cardiovascular function inadequately explains the comprehensive mechanism through which exercise promotes cognitive function. For example, Stanley et al. [26] found that exercises such as stretching and coordination had little effect on cardiovascular function, but can promote cognitive function.

Exploring the complex mechanisms underlying the cognitive benefits of exercise through advanced brain imaging technologies has garnered increasing attention. Previous studies [27] have confirmed that there are spontaneous neuronal activity abnormalities in local brain regions of people living with diabetes, and the functional connectivity of brain networks has also changed. Importantly, such aberrations in brain connectivity and network attributes may emerge as pivotal pathophysiological features of diabetes. In the realm of prolonged exercise regimens, whether involving moderate-intensity aerobic exercise or high-intensity interval training, there is a noticeable improvement in cognitive performance and increased brain activation levels among the elderly [28]. However, a nuanced perspective is essential, as some studies have found a contrasting impact of long-term exercise on brain activation levels in the elderly, for example, after a 12-week intervention, the activation levels of the prefrontal cortex induced by overall cognitive tasks showed a significant reduction post exercise compared with the pre-exercise levels [29].

Exercise has the potential to enhance cognitive function in patients living with T2DM through its impact on insulin sensitivity and the regulation of brain glucose metabolism. The intricate interplay between insulin and glucose metabolism is pivotal in governing cognitive processes. Insulin, concentrated in receptors in the brain, not only orchestrates glucose levels throughout the body but also plays a supportive and protective role in neuronal function. The cognitive function of patients living with T2DM is intricately linked to insulin signaling, as insulin resistance within the central nervous system emerges as a key mechanism underlying cognitive impairment in these individuals. In the realm of brain memory processes, the endogenous insulin receptor in the hippocampus assumes particular significance [30]. Insight from McNay et al. [31] revealed that insulin injection into the hippocampus enhances local glycolysis and augments glucose uptake and metabolism. However, this metabolic boost is notably diminished in animals with T2DM, indicating the impact of insulin resistance. Furthermore, insulin’s regulatory role extends to nerve metabolism, particularly in stimulating the medial temporal lobe and hippocampus, and thereby increasing glucose uptake. By bolstering insulin sensitivity, exercise becomes a crucial intervention, ensuring an ample supply of glucose to the brain, particularly the hippocampus. This appears to be a pivotal strategy in mitigating cognitive decline among patients living with T2DM. The indirect influence of exercise on cognitive function in patients living with T2DM is underscored by its ability to control blood glucose levels. Beyond the direct effects on insulin and glucose, exercise serves as a mitigating force against the deleterious consequences of long-term hyperglycemia. These factors collectively impact the nervous system. Additionally, chronic hyperglycemia leads to structural changes in cerebral vasculature, including thickening of the cerebral vascular basement membrane, reduced vessel count, and diminished cerebral blood volume. This vascular remodeling heightens the risk of local hypoxic-ischemic brain damage, ultimately culminating in cognitive decline [32]. Therefore, the multifaceted benefits of exercise extend beyond insulin sensitivity, encompassing the mitigation of hyperglycemia-related pathways to safeguard cognitive function in individuals living with T2DM.

### Analyzing the regulatory effect of exercise on cognitive function intervention in people living with diabetes

The overall heterogeneity of cognitive function in people living with diabetes with exercise intervention is I^2^ = 95%, signifying that 95% of the total variation is attributed to the real difference in effect size. This suggests that the effect sizes in individual studies are more varied, and the introduction of adjustment variables is needed for a thorough investigation of heterogeneity. When two variables (variable X and variable Y) are influenced by another variable, M, M becomes the moderating variable [33]. The method of exercise, time, intensity, frequency, and cycle are important adjustment variables for the effect of exercise intervention. The heterogeneity of the included studies was investigated. Subgroup analysis showed that exercise mode, time, frequency, and cycle may be important sources of heterogeneity. However, due to the limited number of included studies, it is still impossible to exclude the influence of training intensity, age and gender of subjects, specific sports content, and other factors on the heterogeneity of the study. In addition, in terms of methodological quality, because the intervention is exercise intervention, it is difficult to blind the subjects and coaches. Some studies did not implement blinding or did not explain in the text whether to adopt hidden allocation methods, which may increase the risk of bias and become a source of methodological heterogeneity in meta-analysis.

Intervention Content: The study uncovered significant findings regarding the impact of various exercise modalities on cognitive function. Specifically, physical and mental exercise, aerobic exercise, and multi-component exercise demonstrated significant effects, highlighting the effectiveness of these interventions. Previous meta-analysis found that multi-component exercise intervention was not superior to single exercise in improving the overall cognitive function of elderly patients living with T2DM [34]. The results of this study are inconsistent—multi-component exercise produced a larger effect size and the intervention effect was better. This may be because the number of studies included was small, and subgroup analysis was more confounded by random errors. Recent study have shown that enriched environments and diverse exercise modalities contribute to increased neurogenesis [35]. Multi-component exercise, involving two or more exercise types [36], such as aerobic exercise, strength/resistance training, balance/coordination training, and flexibility training, has gained attention. Some studies have shown more cognitive benefits of multi-component exercise interventions, such as aerobic, resistance, balance, and flexibility training in healthy older adults, especially when combining aerobic and resistance exercises [37, 38]. Research indicates that multi-component exercise surpasses single exercise modes [39] in improving blood glucose control [40], blood lipid levels [41], and reducing inflammatory status in individuals living with T2DM.

Beyond metabolic effects, multi-component exercise induces favorable adaptations in the nervous system through neural plasticity and modulation of various signaling pathways. This comprehensive approach not only enhances cognitive function but also improves attention, language fluency, and overall cognitive abilities in the elderly, showing potential therapeutic effects on cognitive impairment in this population. Aerobic exercise stands out for its ability to improve cardiovascular function in people living with diabetes, enhancing brain tissue perfusion, and inducing remodeling of hippocampal neural pathways. As a result, aerobics has a crucial role in enhancing cognitive function in people living with diabetes [42]. Equally, resistance exercise enhances muscle pumps, leading to peripheral vascular compression and subsequent increases in stroke cardiac output. This cascade effect improves cerebral perfusion and brain-derived neurotrophic factor [43]. However, it is noteworthy that this study did not observe a significant effect of resistance exercise on cognitive function intervention. This may be attributed to a lack of relevant clinical research, resulting in a relatively limited inclusion of literature in this study. Future investigations should prioritize exploring resistance exercise interventions in cognitive function for patients living with diabetes to strengthen the evidence base for comprehensive evaluation.

Intervention Duration, Frequency, and Timing: Optimal Parameters for Cognitive Benefits Results have shown that exercise interventions exceeding 24 weeks yield the maximum effect size. A shorter movement cycle is notably associated with suboptimal intervention effects, potentially due to neurodegenerative changes and other factors [44]. Examining intervention frequency, the results highlight that interventions conducted more than three times a week yield the most significant and effective outcomes. Devore et al. [45], spanning an average of 2 years, observed 1,550 female patients aged over 70 living with T2DM. Using the MMSE to gauge cognitive function, the study revealed that long-term high-level physical activity, adjusted for age and education, correlated with enhanced overall cognitive and verbal memory functions. This underscores the notion that a higher frequency of exercise translates to superior cognitive benefits. Similarly, Colberg et al. [46] found a positive correlation between moderate-intensity aerobic exercise (at least 30 minutes, three times a week, for a minimum of 1 year) and total cognitive function scores in patients living with T2DM, as measured by both MMSE and the St. Louis University Mental State Examination Scale (SLUM). A cohort study by Shih et al. [47] of 1,438 patients living with T2DM revealed that individuals with lower physical activity levels had a heightened risk of dementia or cognitive dysfunction during a 10-year follow-up. The association may be attributed to the reduction of overall sedentary time and the continuous induction of exercise factors through high-frequency exercise, thereby enhancing neurocognitive health. Nevertheless, future studies should delve into the intricate relationship between exercise-induced neurobiological mechanisms and dose parameters. Considering intervention time, our results indicate that the effect of time >40 minutes is most significant, which is consistent with previous studies; longer exercise time may bring greater cognitive benefits to the elderly [48]. The included literature in this study indicates that a maximum intervention time of 65 minutes, with most studies adopting a 60-minute duration, is the most beneficial. Striking a balance is crucial, as excessively long sessions may induce fatigue, compromising the efficacy and enthusiasm of elderly participants. Conversely, overly brief sessions may lack the requisite stimulation for achieving optimal intervention effects. Consequently, the recommended timeframe falls within the range of 40–60 minutes.

Noteworthy Considerations on Intervention Intensity It is noteworthy that 42% of the literature included in this study focused on intervention intensity [49–53]. However, certain literature presents challenges in clarifying the intensity setting. Examples include references to “30 minutes of moderate-intensity walking,” instructions to “determine the intensity according to the subject,” and the use of different color elastic bands to signify various intensities. Compounding this issue is the absence of standardized criteria for intensity, such as “reserve heart rate percentage” and “1RM percentage.” These inconsistencies pose difficulties in systematically extracting intervention intensity data from the literature.

Limitations of the Study While this study rigorously adhered to the guidelines outlined in the PRISMA statement, several limitations warrant acknowledgment. 1) The literature search, though meticulously aligned with PRISMA criteria, omitted unpublished works, and some studies were excluded due to incomplete outcome index data, potentially introducing a degree of data incompleteness; 2) Discrepancies in participant characteristics, including age, gender, and disease severity, pose a potential source of variability that may impact result precision; 3) Several studies lacked blind implementation or failed to elucidate the employment of concealed allocation methods, heightening the risk of bias; 4) With intervention times ranging from 15 to 65 minutes, and a predominant focus on 60-minute sessions, the analysis falls short of elucidating the upper limit of effective exercise duration for cognitive function intervention in patients living with diabetes, thus limiting insights into the sustained impact of prolonged exercise.

## Conclusion

The findings of this meta-analysis reveal that exercise intervention significantly contributes to mitigating the decline in cognitive function in people living with diabetes. The effectiveness is intricately modulated by various variables, and optimal outcomes are observed with multi-component exercise, singular sessions lasting 40–60 minutes, a weekly exercise frequency >3 times, and sustained exercise over 6–12 months. However, it is crucial to approach these results cautiously due to the limited number of incorporated studies and substantial heterogeneity among them. To strengthen the evidentiary foundation, further RCTs featuring standardized study designs are imperative. Moreover, there is a lack of research into the impact of exercise on the specific cognitive functions of elderly individuals living with T2DM, highlighting the need for dedicated attention in future studies.

## Supporting information

S1 Table PRISMA 2010 checklist. (DOCX)
